# European Perspective on How Social Prescribing Can Facilitate Health and Social Integrated Care in the Community

**DOI:** 10.5334/ijic.7636

**Published:** 2023-05-05

**Authors:** Donata Kurpas, Juan Manuel Mendive, Josep Vidal-Alaball, Ferdinando Petrazzuoli, Mohammed Morad, Thomas Kloppe, Wolfram Herrman, Nataša Mrduljaš-Đujić, Joyce Kenkre

**Affiliations:** 1 Health Sciences Faculty, Wroclaw Medical University, Poland; 2 WONCA Europe Social Prescribing and Community Orientation Special Interest Group, Poland; 3 European Rural and Isolated Practitioner Association (EURIPA), Poland; 4 European Rural Isolated Practitioner Association, WONCA Europe Social Prescribing and Community Orientation Special Interest Group, Spain; 5 Institut Català de la Salut, Universitat Autònoma de Barcelona, Spain; 6 WONCA Europe Social Prescribing and Community Orientation Special Interest Group, Spain; 7 European Rural and Isolated Practitioner Association (EURIPA), Spain; 8 Institut Català de la Salut and University of Vic, Cataluña, Spain; 9 WONCA Europe Social Prescribing and Community Orientation Special Interest Group, Sweden; 10 European Rural and Isolated Practitioner Association (EURIPA), Sweden; 11 Department of Clinical Sciences in Malmö, Centre for Primary Health Care Research, Lund University, Malmö, Sweden; 12 WONCA Europe Social Prescribing and Community Orientation Special Interest Group, Israel; 13 European Rural and Isolated Practitioner Association (EURIPA), Israel; 14 Department of family medicine, Community Health Division, Faculty of Health Sciences, Ben Gurion University of the Negev, Israel; 15 WONCA Europe Social Prescribing and Community Orientation Special Interest Group, Germany; 16 Department of General Practice and Primary Care, University Medical Center Hamburg-Eppendorf, Germany; 17 Institute of General Practice and Family Medicine, Charité – Universitätsmedizin Berlin, Germany; 18 WONCA Europe Social Prescribing and Community Orientation Special Interest Group, Croatia; 19 European Rural and Isolated Practitioner Association (EURIPA), Croatia; 20 Department of Family Medicine, University of Split, School of Medicine, Croatia; 21 WONCA Europe Social Prescribing and Community Orientation Special Interest Group, UK; 22 European Rural and Isolated Practitioner Association (EURIPA), UK; 23 University of South Wales, UK

**Keywords:** Social Prescribing, integrated care, primary care, community, needs, assets

## Abstract

Social Prescribing is a mechanism by which primary care team members can refer patients to community groups to improve their health and well-being. It integrates health, social care, and community, allowing patients to actively improve their health and well-being by participating in community initiatives and activities. These activities have traditionally been part of community life in European countries, and the benefits need to be consistently recognized.

## Context and objective of a key process or aspect of integrated care

This perspective paper aims to demonstrate the benefits of social prescribing to add another dimension to integrated care now and in the future. We aim to open the discussion by identifying European community health and social care needs and how members can initiate change to take responsibility for their health and well-being and that of others.

## Nature and role of the author in supporting or influencing care integration

The authors of this perspective are members of the European Rural Isolated Practitioners Association (EURIPA), a network organization founded by family physicians to address the health and wellbeing needs of rural communities and the professional requirements of those who practice in those communities throughout Europe. They have knowledge and experience and have recognized the benefits of social prescribing for developing integrated care.

## Description of the innovation, policy

Social Prescribing (SP) enables people to receive support in their community to meet their social, emotional or physical needs through non-clinical community support. The goal is to improve their mental health and physical well-being by allowing them to focus on what is important to them, thus giving them more control over their health and well-being [1]. It is often described as an innovative form of integrated care more focused on social engagement, as social activities are considered protective factors against isolation and subjective loneliness [2,3,4]. Social prescribing is sometimes known as community referral and by similar names. It occurs through referrals from primary care services to various community groups organized by, for example, community members themselves, the local government, or local charities. The types and topics to which people are referred and benefit from them emotionally and physically include walking, cooking, crafts, fieldwork, beekeeping, group learning, gardening, sports activities, arts and making friends. SP is believed to significantly impact on the social determinants of health by increasing community participation. Developing social capital can improve community health, well-being, and prosperity.

## Reflection - development of the integration of care

### Infrastrucuture

An essential factor specific to SP interventions is the local infrastructure. SP interventions are usually provided by the third sector to deliver activities and has been identified as a factor influencing the success of SP [5,6]. These include various non-government organisations, charities, community groups and volunteers. It has to be considered that there are health systems in Europe similar to those in Germany where the provision of integrated care is almost nonexistent. Until recently, there was a lack of connection between primary health care and social services [7]. Especially where health and social care are organized by different departments and are subject to different laws. However, it is known that most primary care physicians would like to have formal contact with the social care sector to address social problems more effectively [8,9]. To enable some of these required changes will need policy development such as the Well-being of Future Generations (Wales) Act (2015) [10], which sets the goal, permission, and legal obligation to improve social, cultural, environmental, and economic well-being in Wales [11]. The Act was intended to make organizations think more about long-term outcomes to: work better with people and communities and each other; prevent problems and improve access to well-being services and activities; and take a more integrated approach.

## Assessment of need

Social prescribing was first introduced in Catalonia, Spain, in 2012 as a pilot program and has been implemented progressively. One of its main characteristics was that it was explicitly developed from and for primary care and used the Health Assets model [12]. Consequently, before implementing a social prescribing program in an area, health asset mapping must be conducted and is considered necessary to regularly update the community resources being offered. Primary care, public health, social services, and the community must work together to do this. It has been demonstrated that the Social Prescribing program can only be considered successful when this synergy occurs in a specific area [13]. The program has identified gaps to address needs, for example: exercise and social interaction, social interaction and group activities for women and cultural integration for Immigrants. To address the language needs of migrants, libraries have become involved in teaching Spanish.

The pandemic caused by the SARS-CoV-2 coronavirus and its severe impact on the social lives of citizens, especially in terms of loneliness and social isolation, have also led to new social prescription programs to mitigate these effects. In South Korea, for example, during the pandemic, a pilot program targeting the elderly rural population showed that an intervention consisting of musical storytelling, a support group, and gardening could help reduce feelings of loneliness and depression and increasing self-confidence [14].

In Europe, there are several models of social prescribing. In Poland, it is often associated with lifestyle medicine. The prevalence of lifestyle-related diseases in Poland and the distribution of underlying risk factors has generally increased in recent decades, with only a few minor exceptions, such as smoking rates [15]. Consequently, the first social prescribing activities focused on primary prevention measures, mainly increasing physical activity, correcting diet, smoking cessation, and reducing alcohol consumption.

## Barriers to progress

In some countries, including Italy, the term “social prescribing” remains unpopular and is not understood by most health care workers. Some SP activities occur locally, but overall, these could be better organized as in other European countries. Other issues impact the provision of services in the type and quality of services provided between urban facilities in northern Italy and the rural and underserved areas in southern Italy. In the rural areas of southern Italy, it is still strongly linked to the medical sector: Patients who may be eligible for SP are those who use what is called “assistenza domiciliare programmata” (scheduled home care) or “assistenza domiciliare integrata” (integrated home care). Unfortunately, these are not social activities that allow people to learn new skills, extend their abilities, or make new friends.

In Croatia, where there is currently no development of social prescribing, the rural physician often works alone with a heavy workload but realizing there is a need in the community for additional services, such as alcohol abuse treatment. So where do they start? How to engage with the community to address issues often not spoken about may seem daunting. How to coordinate such a new development, solicit feedback to monitor outcomes, and ensure safety? So is this an opportunity to discuss and develop integrated care models for the future facilitated by the development of social prescribing in communities to address their local needs.

## Lack of evidence

The literature continues to need more evidence on the effectiveness of social prescribing [16] and an understanding of intervention mechanisms [17]. It is challenging to quantify specific outcomes for potential changes due to individual circumstances and associated health benefits. Although the effectiveness of social prescribing programs is becoming acknowledged, evaluation is not straightforward as SP programs are not a quick fix to address problems of inequality and social exclusion in disadvantaged areas [18].

Developing social prescribing and working with the community can be an ideal approach to complement and expand opportunities for better health for individuals by contributing to a personalized approach. At the same time, shift the focus from the need for medication to healthier living in their community. For this to happen, working with key people and coordinating activities is necessary, so the GP or other professionals can recommend specific activities and monitor patient outcomes [18].

## Conclusions and lessons that can be drawn

Integrated health services aim to deliver comprehensive care to individuals across their life course by strengthening health care systems to achieve this goal, and social prescribing has been identified as a concept that could facilitate community engagement as SP involves connecting individuals with non-medical sources of support in their community such as volunteer opportunities, exercise programs, or cultural events. Consequently, SP can address issues of social isolation, promote mental and physical health, and enhance community engagement. Therefore, adopting social prescribing can contribute to developing integrated health services by enabling communities to participate in providing care.

An important factor is leadership within and between groups, health, social, and community, organisations, culture flexibility, and resource availability [5]. Another factor is the development of SP service level and partnership agreements, communication strategies and patient engagement. Research has found that factors that enable proper implementation of integrated care include new staff training, staff stability, physician engagement, and information technology systems [19], but barriers and facilitators to the implementation of integrated care are based on organisational change [20].

There are still many challenges facing SP, including funding and sustainability, clarity around the role of SP within healthcare systems, and a need for more evidence around the effectiveness of SP in improving health outcomes. To address these challenges, SP could fit into a community-based integrated care system as one tool among many. This could involve collaboration between healthcare providers, social care providers, and community organizations to address the social determinants of health and improve overall health outcomes [21].

It is essential to acknowledge the criticisms that some have of SP. These include concerns around the potential for medicalising social problems, issues with the quality and consistency of social prescribing services, and questions around the effectiveness of SP in addressing social determinants of health. However, developing the argument for SP by emphasizing its potential to promote better integration and integrated care is crucial. This could involve highlighting the benefits of a holistic approach to healthcare that addresses social determinants of health and supports overall wellbeing [22].

Overall, SP has the potential to evolve beyond its current function to connect with social care. By addressing challenges and collaborating with other healthcare providers and community organizations [23]. SP could be used in various areas and applications to support integrated care and promote better health outcomes. Acknowledging criticisms of the concept while emphasizing the need for further development beyond doctors’ current episodic/medical thinking is essential.

SP must integrate with health and social services to significantly improve population health.

In the words of Anthony D’Angelo

“Without a sense of caring, there can be no sense of community”.
